# What Lies Beneath? Using Point of Care Ultrasound (POCUS) to Identify Soft Tissue Foreign Bodies in Children and Adults: A Literature Review

**DOI:** 10.24908/pocusj.v10i01.18072

**Published:** 2025-04-15

**Authors:** David McCreary, Bhaskar Sarvesh, Maria Munir

**Affiliations:** 1 Paediatric Emergency Department, Sunderland Royal Hospital, Kayll Road, Sunderland, GBR

**Keywords:** POCUS, soft tissue ultrasound, foreign body, foreign bodies, granuloma

## Abstract

**Objective:**

We aimed to evaluate and appraise the existing evidence on the use of point of care ultrasound (POCUS) for identifying soft tissue foreign bodies (FBs).

**Methods:**

We searched PubMed, Medline, CINAHL, and Cochrane databases for prospective and retrospective studies evaluating the reliability of POCUS in identifying soft tissue FBs. Our primary intention was to review the paediatric-specific evidence base. However, due to a paucity of literature in this area, we also included relevant adult studies and case reports.

**Results:**

We identified a total of 42 unique articles with relevance to our study objective, of which 3 were paediatric cohort studies and 5 were cohort studies involving paediatric patients. There were two paediatric case series and six individual case reports relating to paediatric patients. The remaining studies either involved adults, did not specify the age of their subjects, or were relevant in-vitro studies.

**Conclusion:**

POCUS-users regard it as an effective tool for detecting soft tissue FBs. However, the existing evidence base for POCUS use in paediatric patients is limited. Evidence in adults is also relatively lacking compared with other areas of POCUS research, with few large studies evaluating its reliability. This literature review highlights the need for a large prospective paediatric study in order to confirm its effectiveness compared to traditional radiological imaging.

## Case History

**Figure 1.**
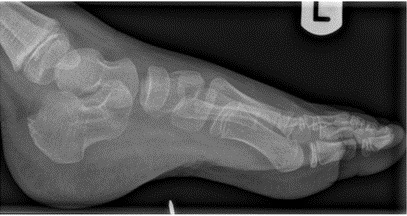
Lateral x-ray of foot

**Figure 2.**
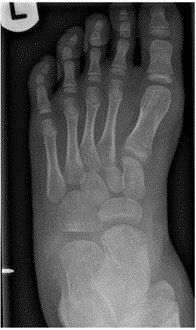
AP x-ray of foot

**Figure 3.**
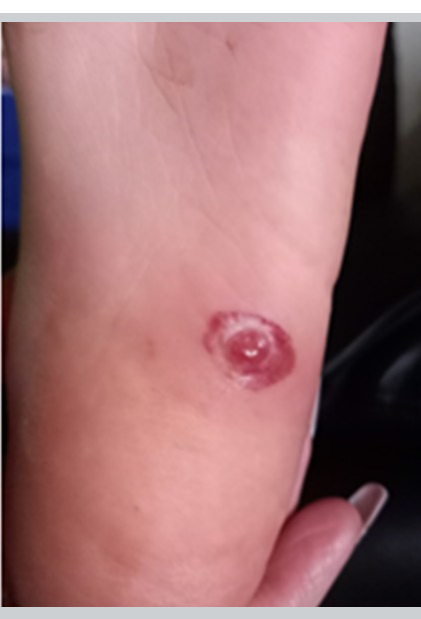
Granuloma on plantar aspect of foot

**Figure 4.**
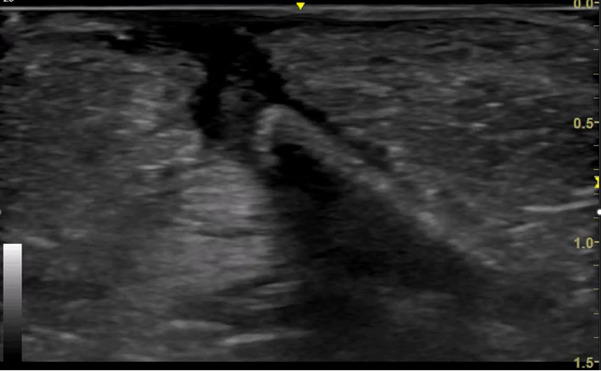
POCUS of soft tissues of foot revealing linear, hyperechoic FB

**Figure 5.**
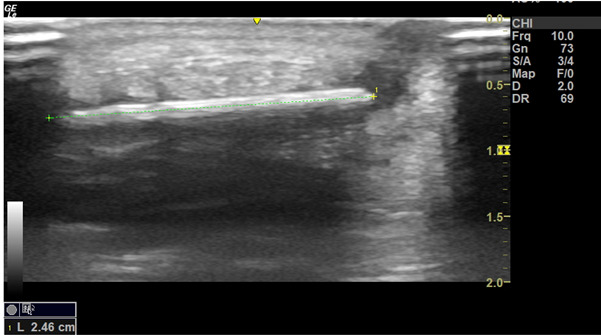
POCUS of linear FB measuring 2.46cm in long axis

**Figure 6.**
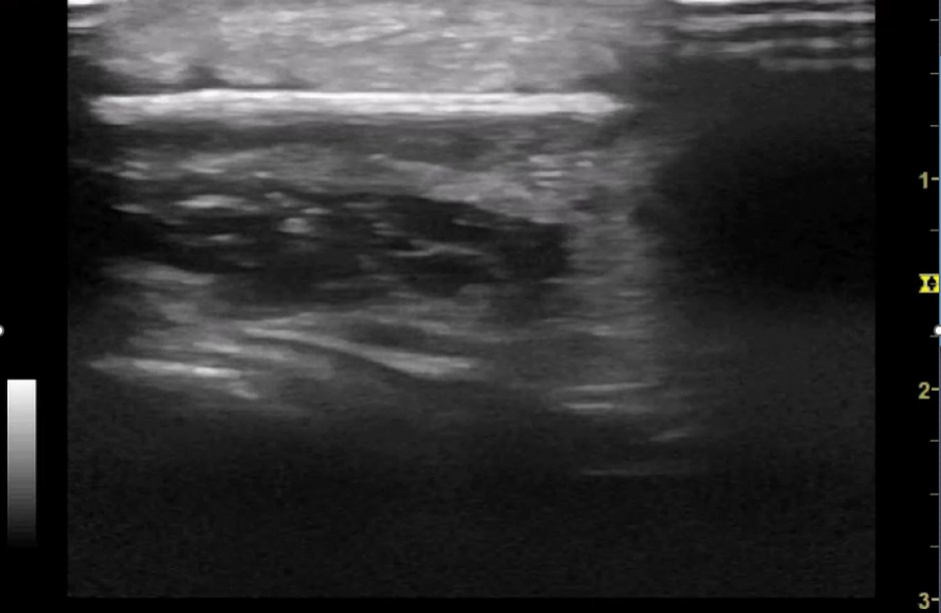
POCUS of foot demonstrating hypoechoic collection indicative of organising seroma deep to FB

**Figure 7.**
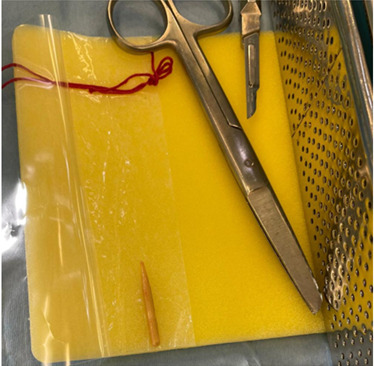
Large piece of wooden barbecue skewer removed from patients foot

A five-year-old boy with no significant past medical history presented to the Paediatric Emergency Department (PED) after standing on something sharp while barefoot in his bedroom. He was walking with a limp, and his examination revealed a 2 mm puncture wound on the lateral plantar aspect of his left foot. Initial X-rays of his foot did not reveal any abnormalities or foreign bodies (FB). The child re-presented two days later with ongoing pain. Again, repeat X-rays did not reveal the presence of a FB (See Figures 1 & 2). Later, 31 days after the initial injury, the child re-presented to the PED after he noticed a strange lesion on the lateral aspect of his foot (Figure 3). The child's ongoing pain and presence of granuloma increased the suspicion of retained soft tissue FB, so one of the authors was asked to conduct a point of care ultrasound (POCUS) examination. This revealed a linear, hyperechoic structure within the soft tissues on the plantar aspect of the child's foot (Figures 4 & 5). There were features of an organizing seroma typified by the hypoechoic collection inferior to the FB (Figure 6). The orthopaedic team were shown images from the child's POCUS, after which the child was taken to theatre. A large piece of wooden barbecue skewer was removed under general anaesthetic (Figure 7). The child was given oral antibiotics post-operatively and recovered well.

This case highlights the importance of conducting soft tissue POCUS to check for FBs at the earliest opportunity. Given the multiple X-rays that were falsely reassuring in this case, the potential for ongoing FBs was overlooked. Owing to its ability to provide detailed, dynamic views in multiple planes—along with its simplicity and safety relative to repeated X-rays—we feel that POCUS is a more appropriate investigation tool for this problem.

## Introduction

Retained FBs have long been a thorn in the proverbial side (or soft tissue) for clinicians. One early study in 1982 concluded that plain X-rays for wooden FB were negative in 86% cases, despite later being present on removal [1]. The earliest literature on ultrasound for soft tissue FBs was published almost four decades ago, where it was suggested as a reliable alternative to X-rays based on studying canine subjects [2].

More recently, in-vitro studies have demonstrated the ability of ultrasound to provide a greater sensitivity compared with X-rays as well as computed tomography (CT) [3,4]. Little evidence exists in relation to POCUS performed for this purpose in emergency departments, and even less relating to paediatric patients. The only existing systematic review of the literature on the reliability of ultrasound for soft tissue FBs was conducted over a decade ago. The meta-analysis published by Davis et al. in 2015 reviewed 17 articles which reported qualitative data, including sensitivity and specificity. They provided an overall sensitivity of 72% (57%–83%) and specificity of 92% (88%–95%) [5].

The purpose of this review is to compile all the existing literature on the use of POCUS for soft tissue FBs in both paediatric and adult patients.

## Methods

**Figure 8.**
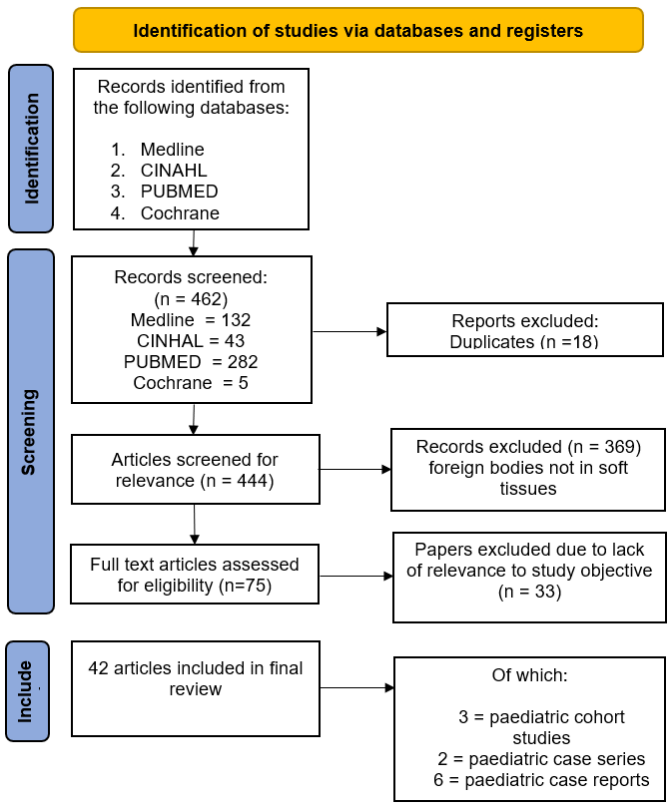
Preferred Reporting Items for Systematic reviews and Meta-Analyses conducted on 23/08/2024.

All three of the authors conducted searches of PubMed, Medline, CINAHL and Cochrane databases for both prospective and retrospective studies evaluating the reliability of POCUS for paediatric soft tissue FBs. Studies were included if they related to POCUS or radiology performed ultrasound and paediatric patients. Due to a paucity of literature in this area, we included studies and case reports relating to both adults and paediatrics. Adult or in-vitro studies were reviewed and appraised if they provided a significant contribution to the existing literature. The full texts of articles were screened by all three authors for inclusion in the final analysis, and there was full agreement regarding which papers to include. (See Figure 8)

The search strategy we used was as follows:

SEARCH TERMS:

AB (“foreign body” OR “foreign bodies”) AND AB (POCUS OR ultrasound OR “point of care ultrasound”)

AB (paediatric OR pediatric OR children OR infant* OR adolescent*)

(“soft tissue*” OR “skin” OR “muscle*”)

## Paediatric Specific Literature

### Paediatric cohort studies

We identified three cohort studies primarily involving paediatric patients.

In their prospective study, Friedman et al. enrolled 105 patients under 18-years-old with wounds at risk for FBs. Participants were assessed using POCUS by a paediatric emergency physician. Across 105 patients, FBs were identified in 12 cases (9.2%) using POCUS. There was no significant difference in its accuracy compared to radiography, suggesting that POCUS may be a suitable initial screening tool for detecting wound FBs. While the authors acknowledged their relatively small sample size among their limitations, they made a case that POCUS performed better for radiolucent FBs such as plastic and organic matter and highlighted the lack of ionizing radiation as an advantage of using this method [6].

A novel paediatric cohort study in 2022 by Chen et al. examined the role of POCUS when combined with methylene blue staining as part of surgical removal of FBs. Methylene blue was injected within the tissue under interrogation to create a strong visual contrast between the FBs and the surrounding tissue during imaging (differing from contrast enhanced POCUS which involves the use of microbubble contrast agents injected into the bloodstream to enhance the visualization of blood flow and tissue vascularity in organs).

They enrolled 80 patients all less than 18-years of age, 11 of whom underwent removal of FBs with the assistance of ultrasound guidance and methylene blue staining. The second group of 69 patients underwent removal of FBs using conventional surgical methods. Paediatric orthopaedic surgeons performed all procedures and undertook the ultrasound scans. There were no significant differences in either the depth of the FB (p=0.078) or the age (p=0.3036) between the two groups. The authors measured the time taken to complete the surgery, number of radiographs taken during the procedure, and incidence of any remaining fragments of FBs post-surgery and incidence of post-operative infections. They demonstrated that the average surgery duration for the ultrasound group was shorter (0.35 ± 0.10 hours) than in the conventional group (0.49 ± 0.50 hours) (p=0.032). The number of radiographs were less in the ultrasound group (1.33 ± 0.34 X-rays) vs. in the conventional surgery group (4.65 ± 1.81 X-rays) (p=0.021). Their study, though limited in terms of the smaller numbers in the ultrasound group, provided evidence for the efficacy of its use along with methylene blue staining in terms of both shortening operating time and reducing radiograph exposure in children with retained FBs undergoing surgical removal [7].

In their 2018 retrospective study, Rothermund et al. described the important contribution made by ultrasound in the detection and removal of ballistic FBs from 61 children and adolescents—aged 5 to 20-years—who had sustained air gun injuries. Imaging was carried out in the interventional radiology department of a large paediatric hospital. Ultrasounds, combined with other radiological imaging such as X-ray and fluoroscopy, were utilized. This was not a comparative study, but one that described the crucial and significant role of ultrasound to the high success rate and safety of FB removal procedures. They determined that ultrasound was highly accurate as an imaging modality and made the removal process more efficient and less invasive [8].

### Paediatric case series

We identified two individual paediatric case series describing the use of POCUS for soft tissue FBs. Pan et al. published a case series of 11 paediatric patients undergoing ultrasound-guided percutaneous removal of FBs, with the intention of determining POCUS effectiveness and any potential complications. They concluded that it is an effective, less invasive, and safe method for extracting FBs in the absence of an open wound, compared with surgery [9].

Varshney et al. described two paediatric cases where POCUS was used to detect previously unidentified FBs in the soft tissues of the thigh and the foot. They used water bath immersion in order to provide an improved acoustic window. This enhanced superficial structures, making the identification of FBs much easier. The study described several other benefits of using water bath immersion during POCUS examinations. These included reduced discomfort by eliminating the need for direct contact between the ultrasound transducer and the patient's skin, and improved image quality due to a reduced distortion of the superficial tissues that can occur when pressure is applied directly during conventional scanning. They concluded that clinical examination is often indeterminate in identifying as well as gauging size or depth of FBs, and that conventional imaging such as a X-rays can fail to visualize them [10].

### Paediatric case reports

We included six paediatric case reports relevant to our objective.

Kourelis et al. published a case report in 2016 describing the rare clinical scenario of a 14-month-old girl who underwent ultrasound-assisted removal of a FB from the cheek following a long standing, fluctuating swelling and erythema secondary to a radiolucent bundle of organic fibers embedded intramuscularly. Ultrasound permitted the “uninterrupted” monitoring during the removal procedure, compared with “blind” surgical exploration, which would necessitate wider incision, longer operating time, and more traumatic dissection. The authors advocated for this as a first-choice imaging technique in every case of a suspected soft tissue FB. They also postulated that ultrasound ought to take an established place both in the emergency department and the Operating Room. Further, they explored if specialists (in this case, otolaryngologists) ought to be comfortable performing ultrasound scans, and highlighted the close teamwork between radiologists. This teamwork was evident in all stages of this child's management and was key to a successful outcome [11].

Binder, Murphy and Constantine described the case of a retained wooden FB in a 9-year-old boy who presented to the PED with pain to the sole of his left foot, having initially stepped on a sharp object at home 3 months prior. This case had obvious parallels to the case presentation in this article. They demonstrated the instrumental role of POCUS in all aspects of the patient's care, from identifying the FB to performing POCUS on the sole of the foot, including using water bath sonography and facilitating an ultrasound-guided posterior tibial nerve block for FB removal [12].

Sheeka et al. described a case report of a 3-year-old boy with a delayed diagnosis of a retained glass FB in the inguinal region, 9 months following the initial penetrating trauma. Following ongoing groin pain and skin indentation, POCUS examination revealed the FB which had migrated from the upper mid-thigh to the inguinal region. The authors highlighted that POCUS can be an effective initial imaging modality for detecting FBs in children, potentially reducing exposure to ionizing radiation [13].

Another case report by Yanay et al. described a child of unspecified age whose wooden FB was initially missed on X-ray imaging. It was subsequently retained in their thigh, which led to severe necrotizing fasciitis. The authors emphasized the challenges in diagnosing non-radiopaque FBs and highlighted the importance of using ultrasound for early detection [14].

In 2015, Gupta et al. published a case report of an 11-year-old boy with a retained thorn in his foot for a period of 2.5 years after his initial injury. After a prolonged period and being misdiagnosed as a soft tissue mass, the authors concluded that in cases with a negative radiograph and clear history of retained FBs, ultrasound is a vital diagnostic tool. They emphasized that ultrasound is a better imaging modality than medical resonance imaging (MRI) or CT scans due to its cost-effectiveness and wide availability. It was further highlighted that retained FBs, such as a thorn, may mimic a variety of bone, joint and soft tissue lesions leading to delayed diagnosis. The authors raised the clinical importance of early detection of wooden FBs because the delay may lead to soft tissue and bone infections [15].

Moake et al. also demonstrated the straightforward and highly effective nature of ultrasound-guided posterior tibial nerve blocks as a means of providing anaesthesia to the sole of the foot to facilitate FB removal for a 16-year-old girl who stepped on a needle. In this case, the FB was initially identified using X-ray. The POCUS-guided nerve block and subsequent procedures were undertaken by a second-year ultrasound fellow. This highlighted that such procedural skills may be undertaken readily by those who have undertaken focused training [16].

## Mixed Paediatric and Adult Studies

One of the earlier studies exploring the effectiveness of ultrasound in detecting soft tissue FBs was by Blyme et al. in 1990. The researchers conducted a blind study using human cadavers and found that ultrasound detected 58 out of 65 FBs—providing a sensitivity of 89% and a specificity of 93% [17].

In 1995, Mattre et al. conducted a study of 20 patients with suspected wooden FBs in their feet. Ultrasound successfully detected 18 out of 20 FBs with their exact position verified through surgical removal. The authors highlighted the potential for false negatives, as demonstrated through two cases in this study that cited the operator-dependent nature of ultrasound [18].

In 1996, Read et al. conducted a retrospective analysis of 98 ultrasound examinations. Ultrasound was performed on the hand and wrist for various conditions, including FBs, with the aim of assessing the efficacy, role, and limitations of diagnostic ultrasound. They included 16 cases of radiolucent FBs and concluded that ultrasound was highly effective in detecting those FBs which were not visible on X-rays [19].

Mohammadi et al. assessed the diagnostic accuracy of ultrasound for detecting and locating non-opaque FBs in soft tissues. The study involved 47 patients with suspected retained FBs, with ultrasonography identifying them in 45 cases. This paper provided detailed descriptions of the ultrasonographic signs that FBs provide, including posterior acoustic shadowing. This was observed in 36 cases, along with halo signs due to abscess or granulation tissue formation in 5 patients. Surgery was performed on 39 patients, resulting in the removal of 44 FBs. The study concluded that ultrasound is an effective tool for detecting and localizing radiolucent FBs in soft tissues, potentially preventing misdiagnosis during initial emergency evaluations [20].

In their 2009 retrospective case series, Callegari et al. described ultrasound-guided removal of FBs from soft tissues. The study included 62 patients aged between 9 and 65-years. A total of 95 FBs were all successfully removed by the same radiologist, and included glass, metal, vegetable matter, plastic, and stone [21].

Bradley et al. conducted a prospective cohort study evaluating the effectiveness of ultrasound-guided extraction of soft tissue FBs involving 287 patients with ages ranging from 5 to 84-years. The majority of the FBs were in the hands and feet, but other sites included the arm, calf, knee, buttock, chest wall, and face. The FBs varied in size from 0.02 cm to 10 cm. A total of 252 patients underwent successful retrieval with no procedural complications, while failure was experienced in 15 cases. The study concluded that ultrasound is a safe and pragmatic approach to extract percutaneous FBs, with an overall success rate of 88% [22].

A study conducted by Tahmasebi et al. aimed to assess the accuracy of detecting radiolucent soft-tissue FBs in the extremities. Between November 2011 and December 2012, patients with clinically suspected soft-tissue FBs and negative radiographs were referred to a radiologist for sonographic examination and localization of the FB. Patients with positive ultrasound findings were then referred for surgical exploration or ultrasound-guided removal. Of the 51 patients who underwent FB removal, 47 had a confirmed FB (including 31 cases of thorn, 12 of wood, 3 of glass, and 1 of plastic). All the standard radiographs were negative. This is comparable with the study results of Anderson et al.1 The study concluded that real-time high-frequency ultrasound is a highly sensitive and accurate method for detecting and removing radiolucent FBs that are challenging to visualize with standard radiography [23].

Polat et al. conducted a retrospective study in 2018 involving 15 patients who presented to the emergency department with wooden FBs in soft tissue. Two were paediatric patients, aged 6-years-old and 12-years-old, and both of whom had a FB in the plantar surface of the foot. The FBs were radiolucent, and the children presented to the emergency department on the 5th day and 1st day since the injury. The wooden FBs were hyperechoic with a surrounding hypoechoic region owing to oedema and inflammation. They concluded that POCUS can effectively measure the length, width, thickness and depth of wooden FBs. They recommended preoperative evaluation of the dimensions and marking the skin along the long axis to reduce incision length and procedure duration, and to minimize the chances of missing FB parts in the soft tissues [24].

A case series in 2017 by Fu et al. involved 12 patients aged 10 to 68-years, and described the successful use of ultrasound-guided soft tissue FB removal even following late presentation. This was not POCUS, and procedures were performed by one radiologist with more than 10 years of experience. The average depth of the FBs was 8.8 mm (range: 3–23 mm). It was also reported that the FB removal procedure was well tolerated by all patients, who experienced no problems or site discomfort after a mean follow up of 22.4 months post-removal [25].

In 2020, Rooks et al. explored the use of ultrasound in the management of ballistic and non-ballistic FB soft-tissue injuries. They emphasized the role of focused hands-on simulation training in implementing successful FB removal practice. They described ultrasound-guided FB removal as safe and minimally invasive, and how this helps avoid complications [26].

Park et al. described the technique of hydro-dissection as a component of ultrasound-guided percutaneous removal of FBs during their case series of 2 males and 2 females aged 10 to 74-years. All FBs were removed in a radiology department rather than in an emergency department setting. The authors highlighted the benefit of ultrasound in the visualization of surrounding ligaments, tendons, and neurovascular. They concluded that the technique is a less invasive and safer method over surgical removal in the operating room [27]. Other small case series describe how POCUS may identify retained soft tissue FBs which were not easily detectable through physical examination alone [28–30].

One study examined the use of power Doppler in 25 patients with possible FBs. They provided a sensitivity of 92% for the overall detection of FBs, with 2 false-positive findings in which discrete FBs were not seen at gross inspection. Inflammation and scar tissue were present at histologic examination. Hypervascularity immediately surrounding the FB was demonstrated on power Doppler imaging in all cases. In this study, scans were performed by one of two experienced musculoskeletal radiologists rather than emergency physicians. The peripheral extremities (feet and fingers) were involved in all cases [31].

In their study of 19 patients—involving 12 children and the remainder adults—Sheils et al. described localization of FBs using POCUS. Similar to water bath sonography, this was facilitated by the use of small standoff pads that were cut for use on small surfaces [32].

### In-vitro studies

Two notable studies provided evidence of the reliability of ultrasound using in-vitro methods.

Turkcuer et al. conducted a randomized, blinded, in-vitro study using 40 chicken thighs with 2 types of non-radiopaque FBs (wood and rubber) and another 40 chicken thighs as part of a control group. The study objective was to contrast three imaging modalities—plain X-ray, soft-tissue X-ray, and ultrasound—in identifying radiolucent FBs in soft tissue. The overall sensitivity, specificity, positive predictive and negative predictive values of plain radiography for both FBs were 5%, 90%, 33%, and 48%, respectively; those of soft-tissue radiography were 5%, 90%, 33%, and 48%, respectively; and those of ultrasonography were 90%, 80%, 81%, and 89%, respectively. They concluded that ultrasound is better than X-rays which are “suboptimal” at detecting nonradiopaque FBs [33]. Their findings were similar to that of Orlinsky et al., who conducted a separate study using chicken thighs [34].

## Discussion

### Advantages of using POCUS

Other than the obvious benefits of being free of radiation, easily repeatable, relatively painless, and not requiring sedation to obtain detailed images, the cost of ultrasound is much less compared to CT or MRI scans [35]. POCUS allows for real-time visualization of the FB so it can be easily targeted while avoiding adjacent structures such as the vessels and nerves. This improves patient safety compared with standard retrieval [36]. POCUS can identify even small fragments of FBs. One study concluded that objects as small as 0.5 mm may be effectively imaged at depths of up to 4 cm [37].

### Limitations of using POCUS

Carneiro et al. also described depth as a limiting factor in identifying soft tissue FBs. FBs become difficult to visualize if they are located 4.0 cm or more beneath the skin surface. They also highlighted the potential for false-positive results, which can occur due to calcifications, fresh hematomas, or air trapped in the soft tissues—all of which appear hyperechoic on POCUS and mimic FBs. Air may also produce acoustic shadowing which can obscure a FB [38]. Shelhoss et al. described the use of POCUS and water bath sonography in excellent detail, describing it as an “indisputable asset,” but also outlining its limitations as it is highly operator dependent [39]. The size of a FB may impact detectability. One study described the training of military paramedics, and a drop of 18% in successful FB identification when the size of the FB dropped from 2 mm to 1 mm [40].

### POCUS as a teachable skill

POCUS has been well demonstrated as a readily teachable skill to clinicians of all grades, even with short periods of focused training [41–42]. POCUS specifically for the detection of FBs has itself shown to be easy to learn and accurate, even when performed by novice users. A 2010 study based in New Zealand described the use of an experimental phantom model-based assessment for emergency physicians and trainees with a range of prior ultrasound experience. They performed a total of 400 individual sonographic examinations. Emergency physicians correctly identified 29 of 30 FBs, and returned sensitivity, specificity, positive predictive values and negative predictive values of 96.7%, 70%, 76.3% and 95.5%, respectively. Trainees correctly identified 60 of 70 FBs and returned sensitivity, specificity, positive predictive values and negative predictive values of 85.7%, 82.9%, 83.3% and 85.3%, respectively. Interestingly, trainees had a higher ability to correctly identify the number of FBs present (36% of cases) than physicians (25% of cases) [43].

### Incorporation of POCUS into clinical practice

**Figure 9.**
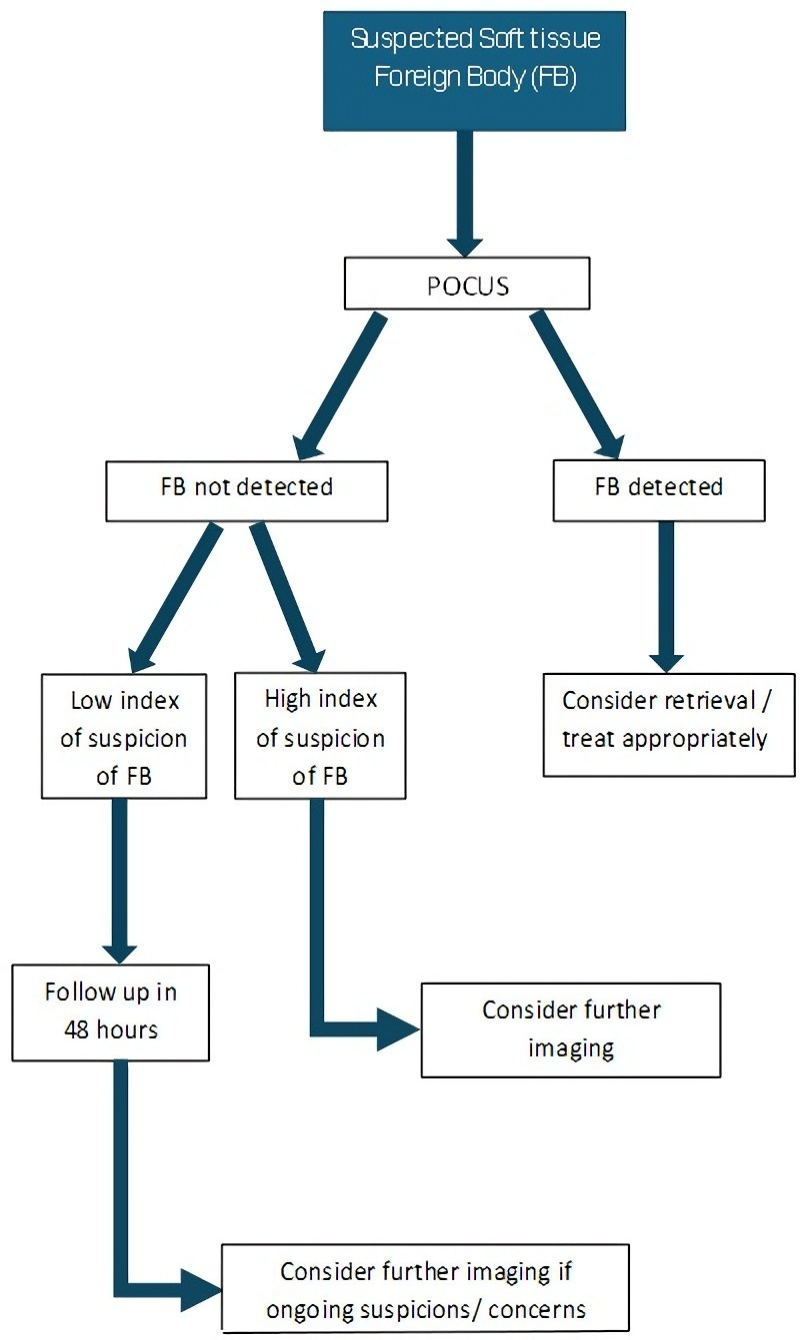
Flowchart for integration of point of care ultrasound (POCUS) into identification of suspected soft tissue foreign bodies (FBs).

Given the impact on patient morbidity, such as cellulitis, osteomyelitis and other complications, as well as the direct effect on the healthcare system from repeat presentations following missed soft tissue FBs, the importance of prompt recognition is clear. X-ray imaging for detecting soft tissue FBs can be unreliable, and paediatric patients can be susceptible to the ionizing effects of radiation. As such, it would seem a natural solution that POCUS be implemented as part of clinical protocols for those presenting to the PED. An example of such a protocol is shown in Figure 9.

This would place POCUS as a first-line evaluation strategy. X-rays or other imaging would follow, and would be considered if sufficient suspicion existed initially or at follow-up visits 48 hours later in cases of lesser concern. Such guidelines should include specific indications, contraindications, and step-by-step guidance on performing and interpreting POCUS. Guidelines should adhere to the governance processes of each institution.

## Conclusion

POCUS for soft tissue FBs has been described as an “indisputable asset,” and users may attest to this. However, the existing evidence base for its use is limited. While smaller studies and case series have demonstrated the utility of POCUS, there is a paucity of evidence pertaining to its use in paediatric patients. Adult evidence is also relatively lacking compared with other areas of POCUS research and has few large studies evaluating its reliability. Given the importance of limiting radiation exposure in children, a large-scale prospective cohort study in paediatric emergency medicine is required to confirm its effectiveness compared to traditional radiological imaging.


