# Supplementary material for: What Lies Beneath? Using Point of Care Ultrasound (POCUS) to Identify Soft Tissue Foreign Bodies in Children and Adults: A Literature Review
Source: POCUS J. 2025 Apr 15;10(1):110–8. doi: 10.24908/pocusj.v10i01.18072 (PMC12057451; doi:10.24908/pocusj.v10i01.18072)
Supplement: Supplementary file 1 [file pocusj-10-01-18072-s001.pdf]

Table of studies included, grouped by study participants (paediatric vs mixed) and study type arranged in chronological order

Primary author	Year of publication	Study participants: Paediatric / Paediatric & adults	Type of study	POCUS / Radiology performed US	No. of patients	Population	Main results	Discussion
Friedman et al.	2005	Paediatric	Prospective Cohort study	POCUS	105	Under 18 years with wounds at risk for FB	Identified 12 cases (9.2%) using POCUS	POCUS performed better for radiolucent FB, highlighted the lack of ionizing radiation as an advantage of using this method
Rothermund et al.	2018	Paediatric	Retrospective Cohort study	Radiology	61	5-20 years old	High success rate and safety of the FB removal	Ultrasound was highly accurate as an imaging modality making the FB removal process more efficient and less invasive
Chen et al.	2022	Paediatric	Cohort study	POCUS (surgeons)	69 surgery<br><br>11 POCUS + surgery	Paediatric	Reduced operating time, reducing radiograph exposure in children with retained FB undergoing surgical removal	FB removal with the assistance of ultrasound guidance and methylene blue staining
Varshney et al.	2017	Paediatric	Case series	POCUS	2	Paediatric age group	Detection of previously unidentified FB in soft tissues of the thigh and the foot using water bath	Clinical examination is often indeterminate in identifying as well as gauging size or depth of a FB, x-rays can fail to visualize them
Pan et al.	2021	Paediatric	Case series	POCUS	11	Paediatric age group	POCUS guided percutaneous removal of FB was effective, less invasive, and safe method for extracting FB in the absence of an open wound compared with surgery	Use of POCUS for percutaneous removal of FB with the intention of determining its effectiveness and any potential complications
Yanay et al.	2001	Paediatric	Case report	Unclear	1	Paediatric-	Wooden FB was initially	Author emphasized the

						Unspecified age	missed on Xray, retained in thigh leading to necrotizing fascitis	challenges in diagnosing non radio-opaque FB and highlighted the importance of using POCUS for early detection
Gupta et al.	2015	Paediatric	Case report	Radiology	1	11 year old boy	With a negative radiograph and unequivocal history of retained FB, ultrasound is a vital part of diagnostic evaluation and a better imaging modality than MRI and CT scan as it is economical and widely available	Retained thorn in foot for 2 and a half years, misdiagnosis of soft tissue mass
Kourelis et al.	2016	Paediatric	Case report	Radiology	1	14 month old girl	Ultrasound assisted removal of a FB from the cheek	Ultrasound permitted the uninterrupted monitoring during the removal procedure compared with blind surgical exploration which would necessitate wider incision, longer operating time, more traumatic dissection
Yhoshu et al.	2020	Paediatric	Case report	Radiology	1	8 year old boy	Ultrasound is cost effective and widely available diagnostic tool, with a sensitivity of 50-90% and specificity of 70-97% for detecting materials like metal, gravel, wood and plastics.	Best modality for detecting retained wooden FB due to the significant acoustic impedance difference between soft tissues and wood
Sheeka et al.	2022	Paediatric	Case report	Radiology	1	3 year old boy	Ultrasound can be an effective initial imaging modality for detecting FB in children, reducing exposure to ionising radiation	A retained glass FB in the inguinal region 9 months following the initial penetrating trauma
Anderson et	1982	Paediatric & adults	Retrospective	Radiology	200	Non Paediatric	In 38%of the patients the	A retrospective review was

al.			cohort			specific	diagnosis was missed by the initial treating physician, in many cases because a roentgenograph of the injured area was not taken	conducted of 200 consecutive patients with FB in the hand seen between 1976 and 1981. Metal was visible in all of the radiographic studies obtained, glass in 96%, and wood in just 15%.
Blyme et al.	1990	In-vitro	Blind study	Radiology	Not specified	Human cadavers	Sensitivity 89%, Specificity 93%	A blind study using human cadavers and it was found that ultrasound detected 58 out of 65 FB providing a sensitivity of 89% and a specificity of 93%
Mattre et al.	1995	Paediatric & adults	Case series	Radiology	20	Non paediatric specific	Ultrasound successfully detected 18 out of 20 FB. There were 2 false negatives	Highlighted the potential for false negatives due to the operator dependent nature of using ultrasound
Read et al.	1996	Paediatric & adults	Retrospective analysis	Radiology	98	Non paediatric specific	Ultrasound was highly effective in detecting those FB which were not visible on x-rays	Ultrasound examination was performed on the hand and wrist for FB with the aim of assessing the efficacy, role, and limitations of diagnostic ultrasound
Turkcuer et al.	2006	In-vitro	Randomised, blinded, in vitro	Radiology	42	Chicken thighs 2 types of FBs rubber and wood	17 out of 20 wood FB (85%) were identified with ultrasonography. 19 out of 20 rubber FB (95%) were by ultrasound.	High frequency ultrasound is better than x-rays which are suboptimal at detecting non radiopaque FB
Callegari et al.	2009	Paediatric & adults	Retrospective case series	Radiology	62	Aged between 9 and 65	A total of 95 FB were all removed successfully	The scans were performed by the same Radiology and FB included glass, metal, vegetable matter, plastic and stone
Mohammadi et al.	2011	Paediatric & adults	Case series	Radiology	47	Suspected retained FB	The study involved 47 patients with suspected retained FB, with	Diagnostic accuracy of sonography for detecting and locating radiolucent FB in soft

							sonography identifying then in 45 cases	tissues
Bradley et al.	2012	Paediatric & adults	Prospective cohort study	Radiology	287	5-84 years old Majority of the FBs were in hands and feet	FBs varied in size from 2 mm to 10 cm. 252 patients underwent successful retrieval with no procedural complications with failure in 15 cases.	It was a safe and pragmatic approach to extract FBs percutaneously using the ultrasound with an overall success rate of 88%
Tahmasebi et al.	2014	Paediatric & adults	Case series	Radiology	51	Unclear FB consisted of 31 cases of thorn, 12 of wood, 1 of plastic	Real time high frequency ultrasound is a highly sensitive and accurate method for detecting radiolucent FB	Aimed to assess the accuracy of in detecting radiolucent soft tissue FB in the extremities
Davies et al.	2015	Paediatric & adults	Systematic review & meta-analysis	Radiology	unknown	Various ages	Sensitivity of 72 % (57-83), Specificity of 92% (88-95)	The meta-analysis reported qualitative data including sensitivity and specificity
Park et al.	2015	Paediatric & adults	Case series		10 to 74 years old	2 Males<br>2 Females	The technique is less invasive and safer method over surgical removal in the operating room	Technique of hydro-dissection as a component of ultrasound guided percutaneous removal of FB
Fu et al.	2017	Paediatric & adults	Case series	Radiology	12	10-68 years old,	FB removal was well tolerated by all patients	Ultrasound guided soft tissue FB removal following late presentation
Polat et al.	2018	Paediatric & adults	Retrospective study	Radiology	15<br><br>13 adults<br>2 children	Mixed ages<br><br>Wooden FB in soft tissue	They described wooden FBs hyperechoic with surrounding hypoechoic region owing to oedema and inflammation.<br>They concluded that US can effectively measure the length, width, thickness and depth of wooden FB	Recommended preoperative evaluation of the dimensions and marking the skin along the long axis to reduce incision length, procedure duration and, minimise chances of missing parts of FB in the soft tissues
Rooks et al.	2020	Paediatric & adults	Training manual	Radiology	Not	Not applicable	Focused laboratory	Sonography provides

					applicable		training is critical to successful implementation of a sonographic FB management practice.	excellent detection, localization, and characterization of soft-tissue FB.
Carneiro et al.	2020	Paediatric & adults	Not specified	POCUS	unknown	unknown	Depth as a limiting factor in using ultrasound to localise soft tissue FB. Difficult to visualize if at 4cm or more beneath the skin surface	Limitations of using POCUS: depth, calcifications, air producing acoustic shadowing
Orlinsky et al.	2024	In-vitro	In-vitro	Radiology	Chicken thighs	In- vitro	Similar findings to Turkcuer et al. who also conducted a separate study using chicken thighs	High frequency ultrasound is better than x-rays at detecting nonradiopaque FB

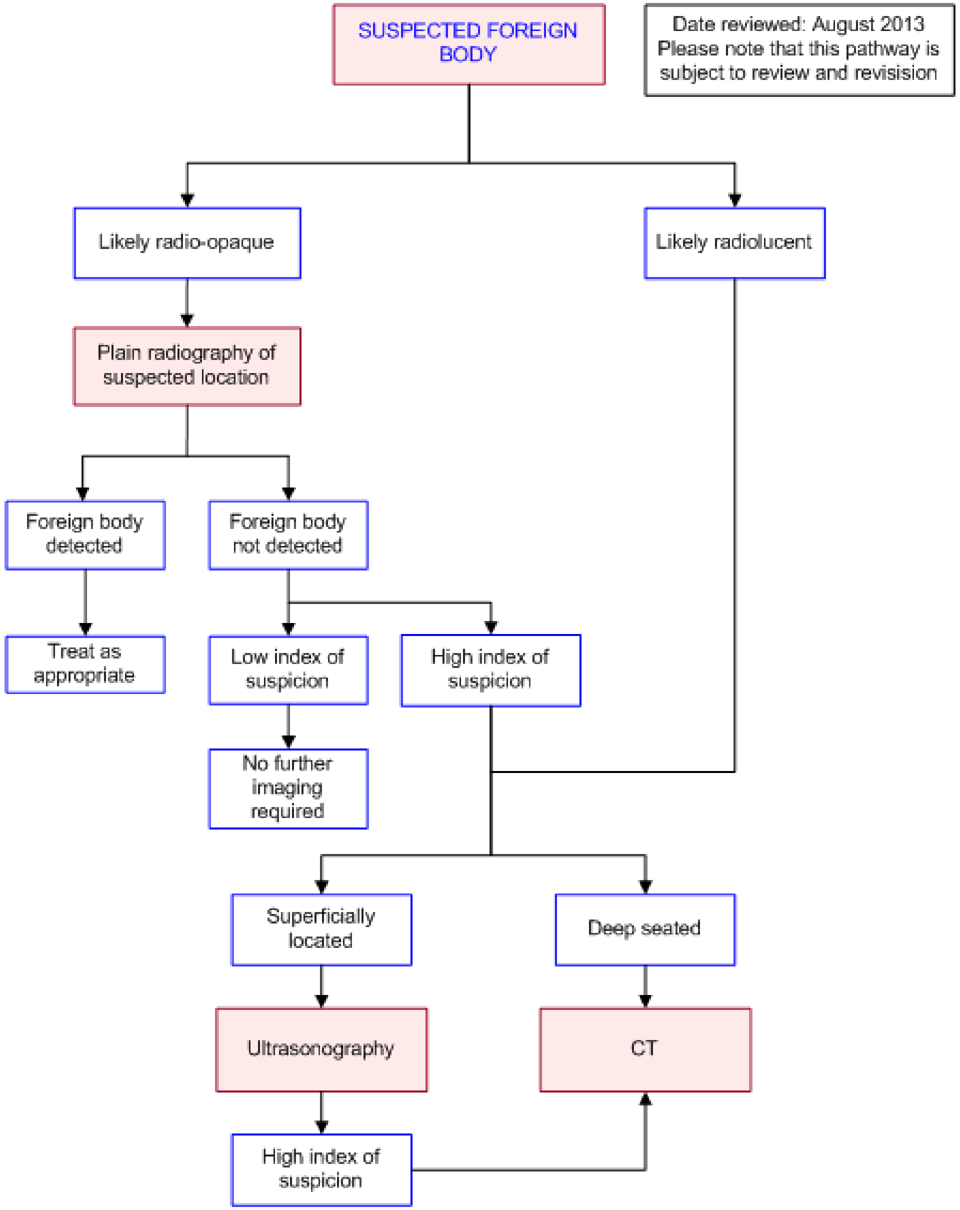
SUSPECTED FOREIGN BODY
Date reviewed: August 2013
Please note that this pathway is subject to review and revisision
Likely radio-opaque
Likely radiolucent
Plain radiography of suspected location
Foreign body detected
Foreign body not detected
Treat as appropriate
Low index of suspicion
High index of suspicion
No further imaging required
Superficially located
Deep seated
Ultrasonography
CT
High index of suspicion
